# Hope, Agency, and the Lived Experience of Violence: A Qualitative Systematic Review of Children’s Perspectives on Domestic Violence and Abuse

**DOI:** 10.1177/1524838019849582

**Published:** 2019-07-01

**Authors:** Lisa Arai, Ali Shaw, Gene Feder, Emma Howarth, Harriet MacMillan, Theresa H. M. Moore, Nicky Stanley, Alison Gregory

**Affiliations:** 1 School of Sport, Health and Social Sciences, 7422 Southampton Solent University, Southampton, United Kingdom; 2 Centre for Academic Primary Care, Bristol Medical School, 1980 University of Bristol, Bristol, United Kingdom; 3 National Institute for Health Research (NIHR) Collaboration for Leadership in Applied Health Care (CLAHRC) East of England, Cambridge, United Kingdom; 4 Offord Centre for Child Studies, 3710 McMaster University, Hamilton, Ontario, Canada; 5 343197 National Institute for Health Research (NIHR) Collaboration for Leadership in Applied Health Care (CLAHRC) West, Bristol, United Kingdom; 6 School of Social Work, Care and Community, 6723 University of Central Lancashire, Preston, United Kingdom; * Author is also affiliated to Population Health Science Institute, Bristol Medical School, 1980 University of Bristol, Bristol, United Kingdom

**Keywords:** children, domestic violence, qualitative, systematic review, services

## Abstract

There is a large body of research on the impact of domestic violence and abuse (DVA) on children, mostly reporting survey data and focusing largely on psychological outcomes. Qualitative research on the views of children has the potential to enable a child-centered understanding of their experience of DVA, so their needs can be better met by professionals. This systematic review reports general findings from the ViOlence: Impact on Children Evidence Synthesis (VOICES) project that synthesized published qualitative research on the experiences of DVA from the perspective of children and young people. A thematic synthesis of 33 reports identified six themes: *lived experience of DVA*, *children’s agency and coping*, *turning points and transitions*, *managing relationships postseparation*, *impact of DVA on children*, *and children’s expressions of hope for the future*. We conclude that professionals working with children affected by DVA should be mindful of the diversity in children’s experiences and listen carefully to children’s own accounts.

## Children and Domestic Violence Abuse (DVA)

DVA (also known as “domestic violence” or “DV” and “intimate partner violent” or “IPV”) is defined as “any incident or pattern of incidents of controlling, coercive, threatening behaviour, violence or abuse between those aged 16 or over who are, or have been, intimate partners or family members regardless of gender or sexuality” (Home Office, 2018). DVA can take physical and emotional forms and includes financial and sexual abuse. Women are more likely to experience DVA than men; around one in four women in England and Wales will experience it in their lifetime (Office for National Statistics, 2017). Many of these will be mothers. It is estimated that 15% of children have been exposed to at least one form of DVA at some point in their childhoods, and 3.1% have been exposed in the last year (Radford et al., 2011).

## Impact of DVA on Children

DVA can damage children’s health and well-being. Several meta-analyses report associations between children’s exposure to DVA and a range of adjustment problems including poor peer relationships and engagement in risky behaviors (Evans, Davies, & DiLillo, 2008; Wolfe, Crooks, Lee, McIntyre-Smith, & Jaffe, 2003). Children exposed to violence have higher rates of physical ill-health and poor-quality sleep (El-Sheikh, Buckhalt, Mize, & Acebo, 2006) and unmet health-care needs such as missed immunizations (Artz et al., 2014). DVA in childhood is also associated with negative health outcomes in adulthood (Russell, Springer, & Greenfield, 2010).

The research evidence on the impact of DVA on children is largely quantitative, focusing primarily on psychological, educational, and, to a lesser extent, physical health outcomes. The earliest systematic review we identified (Fantuzzo & Lindquist, 1989) categorized 29 studies by externalizing and internalizing behaviors as well as by social, intellectual/academic, and physical outcomes. One of the most comprehensive reviews (Artz et al., 2014) reported associations between DVA and a wide range of outcomes, including neurological disorders, delinquency/crime, and victimization, as well as academic and employment status.

While these reviews of epidemiological studies highlight risks to children exposed to DVA, it has been argued that the quantitative literature is dominated by a “…medical/pathological discourse that portrays children experiencing domestic violence as passive recipients of potentially traumatic experiences” (Överlien, 2016, p. 680). In common with other childhood theorists (Callaghan, Alexander, Sixsmith, & Fellin, 2016), Överlien argues that the image of the passive child, positioned as a “bystander” to DVA, is at odds with children’s presentation of themselves as agentic in qualitative research. This tension is reflected in changing the use of terminology in this field with greater use of the word “experience” of DVA rather than “exposure” to DVA (Överlien & Hydén, 2009).

Qualitative research eliciting children’s own experiences of DVA has the potential to give a deeper, more nuanced, and child-centered understanding of what it is like for children to live with DVA, and how it impacts them in ways broader than (although inclusive of) psychological impact.

To date, two qualitative systematic reviews of children’s experiences of DVA have been published. Hines (2015) examined children’s coping, drawing on 17 primary studies to describe nine overarching themes, some of which highlight the tension between children’s agency and passivity. Hines described a variety of strategies used by children experiencing DVA and notes, in particular, their adoption of two roles: *protector* and *victim*.

In the second review (Ravi & Casolaro, 2018), analysis of qualitative studies published after the mid-1980s produced four themes: *context of the abuse*, *immediate reactions to the abuse*, *sequelae reactions*, *and coping*. Coping strategies were described as ranging from “integration” to “distancing.” The authors maintain that children with an “integrating” strategy to DVA may normalize or minimize its impact or even incorporate violence into their own behaviors.

These reviews provide valuable insights into the range of children’s experiences, but each has limitations. In the Hines review, only papers published in the period 1991–2012 were drawn on, a limited number of databases were searched and the studies reported *both* child accounts and adult recollections of childhood experiences. Adult recollection may be compromised by recall bias (Gil-González, Vives-Cases, Ruiz, Carrasco-Portino, & Álvarez-Dardet, 2007); the past is viewed through an adult lens, which can alter how childhood experiences are interpreted. While focusing on children’s accounts, Ravi and Casolaro’s (2018) review drew on only nine papers (from an initial pool of 2,461) from the United States and Europe and only reported the experience of older children (8–17).

Thus, there remains a need for a systematic review of qualitative research on children’s experiences of DVA that focuses on the voices of children, allows for a broad range of impacts (beyond psychological), includes evidence from a wider time span and range of sources, and includes the experiences of children of all ages.

## Responding to the Need of Children Who Have Experienced DVA

In the UK, an NHS England report on improving the response to children’s and young people’s mental health needs noted the importance of improving care of vulnerable groups, including those who have experienced DVA, and identified the value of professional training on exposure to trauma for those working with families (NHS England, 2015).

Interventions such as Identification and Referral to Improve Safety (IRIS; http://www.irisdomesticviolence.org.uk/iris/) in the UK (Feder et al., 2011) and Women who have Experienced Intimate Partner Violence (WEAVE; https://www.weaveinc.org/) in Australia (Hegarty et al., 2013) have been developed to help families impacted by DVA. A wide range of interventions have been developed specifically for children who have experienced DVA, both for children on their own and for children with their nonabusive parent (Howarth et al., 2016). Such interventions are delivered by statutory services and nongovernmental organizations, and the evidence base for their effectiveness is still developing. Those planning and delivering these programs can benefit from enhanced understanding of how children experience DVA abuse and what can enhance their resilience in the face of this experience.

## The ViOlence: Impact on Children Evidence Synthesis (VOICES) Study

The VOICES study, informed by earlier reviews, aimed to produce a child-focused account of children’s experiences. VOICES had two aims: first, to synthesize existing qualitative evidence on the experiences of children living with DVA, drawing on the voices of children themselves; second, to draw out implications for professionals’ responses within health care and other sectors. The present article has a general focus; the aim here is to provide an overview of findings. VOICES’ second aim will be the focus of a later paper, though general recommendations are made below.

### Method

### Review Stages and Inclusion Criteria

The VOICES review comprised of two stages: Stage 1 (mapping), where all studies reported in journal papers, reports, and PhD theses meeting inclusion criteria were identified, retrieved, and subjected to simple descriptive analysis; and Stage 2 (focused synthesis), where a smaller number of studies identified at first stage were subjected to critical appraisal, full data extraction, and synthesis. Prior to both stages, we engaged in a scoping exercise to identify existing reviews and collate examples of terminology used for DVA.

In Stage 1, studies were included which focused on children and young people’s experiences of DVA between parents/carers in domestic settings. To be included, qualitative, *verbatim*, English language accounts provided by research participants aged 18 or under had to be reported in the study. No date or geographical limits were applied. During Stage 2, additional inclusion criteria were applied: sufficient verbatim text from children and young people to contribute to detailed qualitative analysis (i.e., more than two lines of children’s verbatim text) and publication in a peer-reviewed journal.

### Searches

We drew on an earlier search strategy (the IMPRoving Outcomes for children exposed to domestic ViolencE [IMPROVE] project—see Howarth et al., 2019). This strategy included a search of 13 electronic bibliographic databases including MEDLINE, EMBASE, PsychINFO on OvidSP platform, and Cumulative Index to Nursing and Allied Health (CINAHL). We found 8,763 records after de-duplication. One hundred six references potentially relevant to the VOICES review were selected for further screening. We searched for MeSH and text word terms for <Children and adolescents> combined with MeSH and text word terms for <domestic violence>. We combined these with text word terms for <exposure of children to domestic violence or witnessing or growing up with domestic violence>. We updated the IMPROVE search (to April 2016) and also searched: the gray literature; key websites; the reference lists of included studies; and contacted experts for additional materials.

### Screening and Selection of Studies, Critical Appraisal, and Data Extraction

Five authors (L.A., A.G., A.S., E.H., and T.M.) were involved in screening titles, abstracts, and full text papers. The same authors were involved in critical appraisal and data extraction, with two reviewers examining each item, independently and in duplicate. Differences were resolved through discussion. We did not use critical appraisal for exclusion of articles but to describe reporting quality. Titles and abstracts were screened against the criteria described above. Those considered relevant by two reviewers were selected for full paper screening. Eligibility of full text papers was assessed independently by two reviewers.

A predesigned data extraction template was used, which collected descriptions of the reported study, the themes, subthemes, and relevant verbatim quotes from research participants and reviewer ratings of the paper quality based on the Critical Appraisal Skills Programme (CASP) tool for appraisal of qualitative research (CASP, 2017). Data extraction templates were completed independently and in duplicate by two reviewers for each item.

### Synthesis

The synthesis process was led by one author (L.A.) with detailed discussion with two others (A.S. and A.G.). Given the number of papers in the focused sample, we chose a thematic approach to synthesis (Thomas & Harden, 2008). All themes and subthemes, and a sample of data for each theme, were entered into an Excel spreadsheet and then aggregated and reaggregated into final overarching themes and subthemes, with supporting *verbatim* quotes. The process of reading, comparing, regrouping, and reinterpreting data across studies drew on the process of translation used within meta-ethnographic synthesis (Noblit & Hare, 1988) and is similar to the method used by Ravi and Casolaro (2018).

### Findings

Overall, the Stage 1 (mapping) produced 77 items (56 studies identified in the IMPROVE study met VOICES inclusion criteria). An additional 13 items were added from an updated database search. The key website search produced 6 items, and 2 items were identified by word of mouth. Details of the full search strategy and results can be obtained from the first author.

Participants’ age range was 3–25 years (some papers reported analysis of data from young people older than 18 years, which also included data from respondents under 18. We included these but prioritized accounts by under 18s). Most articles were written by authors based in the United States (25) or UK (21). Other researchers were based in Canada, Sweden, Israel, and Norway. Very few items focused specifically on the impact on children’s health/well-being; around a third had a general focus on children’s experiences of DVA, and a smaller number (11) reported children’s experiences of interventions. Most authors had collected data in face-to-face interviews, and thematic analysis was widely used.

Applying stricter inclusion criteria for Stage 2 (described above), we identified a final sample of 33 papers. Most (15) of these were focused on experiences more generally, 5 focused on coping, and the rest (13) included papers with a focus on recovery from DVA (1), health impacts of DVA (1), and postseparation contact (1). Most papers (26 of 33) were rated as being of high quality. However, many did not provide sufficient information about the relationship between the researcher(s) and the research participants (CASP, 2017), and data analysis was often not described in detail (although these deficiencies may be largely a matter of reporting rather than study quality per se). For further information about included papers, see Table 1.

**Table 1.** Details of Articles Included in the Review (Second Stage Only).

Author and Year	Focus	Respondents: Age,^a^ Gender, and Sample^b^	Recruitment and Location	Main Data Collection and Method of Analysis^c^	Quality Appraisal^d^
Aymer (2008)	Coping strategies	14–17, male, *N* = 10	Social services, New York (USA)	Interview, content	High
Benavides (2012)	Spirituality	13–16, mixed, *N* = 14	DVA services, USA	Interview, combined approach	High
Bennett (1991)	General experiences	15–24, female, *N* = 5	Women’s shelters/other Newfoundland (Canada)	Interview, phenomenological	High
Berman (1999)	Health impact	10–17, mixed, *N* = 16	Community sources, Ontario (Canada) and USA	Interview, narrative	Medium
Bertram, Hall, Fine, and Weis (2000)	General experiences	Adolescents, female, *N* = 18	Community center, New York (USA)	Interview, participant observation, thematic	Medium
Bowyer, Swanston, and Vetere. (2015)	Living in temp. accommodation	10–16, female, *N* = 5	Two charities, England	Interview, phenomenological	High
Buckley, Holt, and Whelan (2007)	Impact of domestic violence abuse	8–17+, mixed, *N* = 22	Refuge/other, Dublin (Ireland)	Focus group, analysis method unknown	High
Callaghan, Alexander, Sixsmith, and Fellin (2016)	Sibling relationships	9–11, mixed, *N* = 2 (case studies, three children)	Multisite study	Interview, interpretive interactionism	High
Cater (2007)	General experiences	8–12, mixed, *N* = 10	Women’s shelters, Sweden	Interview, conceptual abstractions	High
Cater and Forssell (2014)	Care by fathers	8–10, mixed, *N* = 10	Women’s shelters, Sweden	Interview, content/other	High
Chanmugam (2015)	Coping strategies	12–14, mixed, *N* = 14	DVA shelters, Texas (USA)	Interview, thematic/other	High
Dryden, Doherty, and Nicolson (2010)	General experiences	12–13, mixed, *N* = 2	DVA services, England	Interview, analysis method unknown	Medium
Ericksen and Henderson (1992)	General experiences	3–10, mixed, *N* = 12	DVA networks, Vancouver (Canada)	Interview, phenomenological	High
Georgsson, Almqvist, and Broberg (2011)	General experiences	8–12, mixed, *N* = 14	Children’s treatment program, Gothenburg (Sweden)	Interview, thematic	High
Goldblatt (2003)	Coping strategies	13–18, *N* = 21	Social services, Israel	Interview, thematic content	High
Joseph, Govender, and Bhagwanjee (2006)	Coping strategies	8–12, *N* = 5	Welfare agency, South Africa	Interview, phenomenological	High
Katz (2014)	Homicide of mothers by fathers	4–7, mixed, *N* = 7	Criminal investigation, Israel	Forensic interview, thematic	High
Katz (2015)	Recovery and the future	10–21, *N* = 15	Voluntary organizations, Midlands, England	Interview, framework	High
Makhosazana Kubeka (2008)	General experiences	14–20, mixed, *N* = 23	High school, South Africa	Focus group, interpretive	High
McDonald et al. (2015)	Animal abuse	7–12, *N* = 58	DVA agencies, Colorado (USA)	Interview, thematic	High
Morrison (2015)	Postseparation contact	8–14, mixed, *N* = 18	DVA services, Scotland	Interview, thematic	Medium
Överlien (2011a)	Experiences of an intervention	5–17, mixed, *N* = 6	Women’s refuges, Norway	Interview, grounded theory	Medium
Överlien (2011b)	Recovery and the future	11–17, mixed, *N* = 5	Women’s refuges, Norway	Interview, ethnographic	Medium
Överlien (2014)	General experiences	12–18, mixed, *N* = 4	Women’s refuges, Norway	Interview, narrative	High
Överlien (2013)	General experiences	8–20, mixed, *N* = 10	Women’s refuges, Norway	Interview, thematic	High
Överlien (2016)	General experiences	8–20, mixed, *N* = 25	Women’s refuges, Norway	Interview, thematic	High
Överlien and Hydén (2009)	Coping strategies	12–15, mixed, *N* = 7	Women’s refuges, Norway	Interview, discourse/content	High
Peled and Edleson (1992)	Experiences of an intervention	4–12, mixed, *N* = 30	DVA project, Minneapolis (USA)	Interview, observation, content	High
Peled (1998)	General experiences	10–13, mixed, *N* = 14	Community/DVA projects, St Paul and Minneapolis (USA)	Interview, content	High
Phillips and Phillips (2010)	General experiences	10–17, female, *N* = 20	DVA services, USA	Participant observation, analysis method unknown	Medium
Stanley, Miller, and Richardson Foster (2012)	General experiences	10–19, mixed, *N* = 19	DVA services, England	Focus group, grounded theory	High
Swanston, Bowyer, and Vetere (2014)	General experiences	8–13, mixed, *N* = 5	DVA charity, UK	Interview, phenomenological	High
Thompson (2011)	General experiences	6–7, mixed, *N* = 4	Child counselling group, USA	Retrospective case study, analytic induction	High

^a^Exact age of respondents could not always be ascertained. In some cases, under 18’s data could not be differentiated from that of over 18’s. ^b^Sample size of child and young people respondents (where this could be ascertained). ^c^Methods refer to those used when collecting data from child and young people respondents (where these could be identified). ^d^Yes = 3, don’t know/to some extent = 2, no = 1, highest quality = 27 (9 × 3), lowest = 9 (9 × 1), range = 9–27. Categorized: 27–22 = high, 21–16 = medium, and 15–9 = low.

Synthesis of qualitative data from the 33 papers included in Stage 2 identified six overarching themes: (1) *lived experience of DVA*, (2) *children’s agency and coping*, (3) *turning points and transitions*, (4) *managing relationships postseparation*, (5) *impact of DVA on children*, and (6) *children’s expressions of hope for the future*, see Table 2. We present each theme below, drawing on the original verbatim text and using the pseudonym allocated by the researcher.

**Table 2.** Critical Findings.

Qualitative research has the potential to illuminate salient aspects of children’s experience of domestic violence and abuse (DVA). From a sample of 33 papers, we identified six themes in the qualitative literature on children and DVA. These included the major themes: “lived experience of DVA” and “children’s agency and coping.” Other themes include one focused on the impact of DVA.

#### Lived experience of DVA

This theme is about children’s everyday lived experience of DVA. It focuses on children’s understanding of the nature of the violence, its severity and frequency, and the family and wider context within which DVA occurs.

##### Living with DVA/nature of DVA

Children describe experiencing different forms of violence. Shouting between parents, angry episodes, and frequent arguments are commonly reported across all review papers. Some children describe witnessing items being thrown or furniture being overturned. Kim (adolescent female), for example, in Bennett’s (1991) study, tells the interviewer how the supper table was upturned by her father and: “…we were sitting there with food all over us…and we were crying and we were really frightened…” (p. 435).

Many children report angry, verbal encounters as well as acts of physical abuse:

…he’d [perpetrator] shout at my mum all the time…get angry—nasty—he never used to do anything like be nice—just kick everyone up the bum or a smack on the back of the head…I got smacked at the back of the head and a kick…and he used to nip your ear…. (Adam in Dryden, Doherty, & Nicolson, 2010, p. 194)

These incidents could become increasingly dangerous and involve controlling behavior, as Kate (aged 8–13) in Swanston, Bowyer, and Vetere’s (2014) study says:

Mum and him [perpetrator] and us…got a house together and after about two weeks he started hitting her. He hit her first because her dinner wasn’t…warm…then he started doing it more and more often. Eventually he tried to stab her. (p. 188)

In her study of children who had experienced extreme forms of DVA and were very fearful as a result, Överlien (2013) draws on Christopher’s (age 16) account as an example of what she calls the “bizarre” acts that sometimes accompany DVA:

Once Mom was on her knees and he [their stepfather] held a gun to her head, and then he asked my little sister if he should kill her or not, and she [the little sister] said no, so she got to choose. (p. 281)

Katz’s (2014) analysis of child (age 4–7) witness accounts of mothers’ deaths at the hands of abusive fathers highlights the ways in which even very young children process extremely violent situations. One child (Interview A) sat patiently outside the room where her mother was being killed by her father, waiting “…for it to end” and, after entering the room, “…saw Daddy sitting on Mommy and putting the knife in her tummy” (p. 1980). Other children report seeing their father strangle their mothers.

Other types of violence reported by children included economic and financial abuse. Aymer (2008) notes that six adolescent respondents (in a sample of 10) had witnessed their parents fighting over money and that family money problems sometimes had serious consequences. Winston, for example, said that “Sometimes there was not enough money to buy food, so we was hungry sometimes” (p. 659).

##### Family and wider context

Some authors situate the child’s experience of DVA in the context of an already troubled family or a neighborhood setting where violence was commonplace. In Phillips and Phillips’s (2010) study, for example, the authors observe that DVA, “…is only one experience within the context of their [the respondents’] lives and is only one aspect of their identity” (p. 308). Caitlin reports that “…I had other things, too. It wasn’t like only DV on me. Like my dad beat me, and my uncles were always killing people” (p. 308). Similarly, Aymer’s (2008) New York–based male adolescents’ accounts are replete with stories of everyday violence in the family, home, and wider neighborhood. Aymer’s adolescent male interviewees also describe parental substance misuse (“…He was high most of the time [and] he was scary to look at…. ” Juan in Aymer, 2008, p. 659), as do several other authors. A boy in Cater’s (2007) analysis of children’s (age 8–12) views of their abusive fathers tells the interviewer his father arrives home at the weekend but then: “…he gets drunk. Then he has a hangover the next day…and then he gets angry at almost everything…He’s rather unfair” (p. 46).

#### Children’s agency and coping

Children’s agency is the focus of this theme, and the myriad ways in which children respond to, and cope with, DVA are able to protect themselves and others (including animals). Children report employing a diverse array of tactics and strategies to manage DVA which can vary according to factors such as the child’s age and the presence of siblings.

##### Actions during DVA

Several authors attempt to categorize children’s actions while DVA is occurring. Georgsson, Almqvist, and Broberg (2011) identify four: shying away, interrupting, watching, and being obstructed from participating. Use of the second of these tactics can mean children putting themselves at risk of verbal or physical assault, such as that experienced by an 11-year-old girl who says that, during violence between her parents, she: “…used to stop them…I pushed dad away…He got mad at me too and told me to shut up” (p. 123). Similarly, Neliswa (aged 18), in Makhosazana Kubeka’s (2008) analysis of data from Black South African adolescents, would sometimes: “…end up being beaten” when she intervened to prevent violence (p. 289).

Chanmugam (2015) categorizes coping strategies during DVA as *problem-solving* and *palliative*; the latter aims to deal with the emotional fallout of DVA but does not modify or resolve the violence itself. Covering ears, getting mad, crying, and refusing to eat are all cited as coping responses. The behavior of 13-year-old Serena to violence (“…I just went into shock and stood there and couldn’t move. But usually I just would hold my head because I’d get a headache and the room would start spinning…” p. 108) is, according to Chanmugam, a response to “palliate emotions.”

Authors’ attempts to classify children’s actions during DVA is a reminder that understanding children’s responses during DVA is not straightforward. What appears to be a passive response to DVA may, in fact, represent a dynamic strategy for dealing with a volatile or dangerous situation (Överlien, 2016). Many children report turning music on or up or wearing headphones to mask the noise of violence, for example. In Överlien and Hydén’s (2009) study, Dina describes how she turned on music because “…when dad got angry we all ran to our rooms…but we knew he would come…and shout terrible things…so I always put on music so I couldn’t hear him shouting” (p. 484). Or children might seek out rooms in the house where the noise of violence was less audible: “…I used to go down to the basement to my big sister because she had her room there…You couldn’t hear as much there. And then we turned the music on” (10-year-old girl, Georgsson, Almqvist, & Broberg, 2011, p. 123).

Children taking comfort from toys might also be seen as a meaningful, context-specific strategy for dealing with violence. Bowyer, Swanston, and Vetere (2015) describing their female respondents (aged 10–16) use of toys or “transitional objects” during DVA, note that these were so important to the girls that “…they would risk their lives to keep this continuity in a context of change…” (p. 314). Ashley (14-year-old female) says that:

If I got worried or upset…I still had my little teddy, which is up in my room, it’s a little dog…I used to hug it, if I got worried or scared…I would basically risk my life just to go and get it. (p. 314)

##### Protecting others

Children’s stories of actions during violence show clearly that, while they sought to minimize harm to self, many also actively attempted to protect others such as siblings and mothers as well as animals. This is an area where age differences are apparent: older children often report adopting a parent-like role in relation to younger siblings and also to mothers.

In Callaghan and colleagues’ (2016) analysis of sibling’s accounts of coping, 11-year-old Rachel describes actions that are highly protective of her brother aged 7:

We were trying not to think about it [the violence]…my brother would think of a game and we’d just start playing it…but we’d still hear the shouting…when the shouting got really loud my brother would just like pause for a minute and look at me ((mimics frightened look)), I’d be like, “It’s OK, it’s OK,” cause sometimes he’d just like freak out and stuff like that…and you’d just…get on with the game quite quickly…carry on playing, make the game like amusing and stuff so he could try and forget about it. (p. 10–11)

The older children in Överlien (2016) report shielding younger siblings from violence; the younger children describe older brothers and sisters distracting them during violent episodes by reading or singing to them or hiding them from danger. Katz (2014) notes that some of the respondents in her very young (4- to 7-year olds) sample (witnesses to their mother’s death) took responsibility for even younger siblings by, for example, removing them from scenes of violence. One child (Interview C) tells the interviewer:

After a few songs, there was a silence so we got outside and saw Mommy with blood, and Daddy…told me I killed Mommy and now I will kill myself, go to your room, so I took the baby and went back to the room. (p. 1980)

Many children describe intervening to shield mothers from abuse: “My mum’s come up screaming…so I’ve locked my bedroom door to keep her in there so he [perpetrator] don’t hurt her” (Kate, 8–13 years old, in Swanston, Bowyer, & Vetere, 2014, p. 190). Supporting mothers was also a role children engaged in when violence was not occurring meaning that, for some respondents, the parent–child relationship was reversed. Jodie in Stanley, Miller, and Richardson Foster (2012) says that:

I used to have my mum crying on my shoulder, now isn’t it supposed to be the other way round?…Whereas I had my mum sat on the stairs, crying on my shoulder at four year-old asking me what she were going to do…I don’t know how to deal with situations at four years old and that’s why it makes an impact, you end up more mature. (Focus Group 3, p. 196)

The abuse of animals in the context of DVA, and children’s efforts to protect pets, is the focus of one paper. A 9-year-old boy in McDonald et al. (2015) states that “When I see my dad is mad, I will take our bird out of the cage and put him in my room. Because I know he will pick the feathers out” (p. 122).

##### Gender differences in dealing with DVA

Gender differences in actions during DVA are discussed in a small number of papers. In a case study of two boys (aged 12 and 13; Dryden et al., 2010), the “heroic protection discourse” (HPD) is utilized to explore gendered understandings of violence. HPD refers to: “…a set of interpretative resources and practices that normalize a form of masculine identity that combines physical strength and aggression with the motivation to use physical force in the service of protecting others” (p. 194). The analysis shows how the interviewees operate with traditional notions of masculinity and femininity, with one child expressing a belief that, had their grandfather been present when their mother was being attacked: “…he wouldn’t have let it happen…he would have killed him” (Adam, p. 193).

Phillips and Phillips (2010) also discuss gender and DVA and observe that the DVA advocates they interviewed: “…often inadvertently reinforced stereotypical gender norms in their everyday, casual interactions with the children” (p. 298), whereas their adolescent, male participants resisted professional notions of the relationship between their gender and DVA: “…the boys…actively resisted the ‘DV stereotypes’ and claimed that they did not fit the intergenerational model…the boys argued that the men who abused their mothers were born violent” (p. 303).

#### Turning points and transitions

This theme captures children’s reports of the points at which their experience of DVA changes, usually by escaping it and taking refuge in temporary accommodation. In one paper (Katz, 2014), the abuse is ended through the death of a mother at the hands of an abusive father.

Escaping DVA was not easy or straightforward. Stanley and colleagues (2012) note that barriers to disclosure include stigma, shame, and embarrassment and that children are aware of this and can be anxious about the consequences of disclosure. Bill, in a young person’s focus group, says: “I was getting like put down at school as well, and loads of people knew about it” (Stanley, Miller & Richardson Foster, 2012, p. 194).

Despite the shame, children describe the ways in which they, alone or with others, reached out for help to end violence. Aranya (8-years-old, female) in Överlien (2016) sought help from a family member when her father began abusing her mother:

When he got angry with mom…I locked myself into the bathroom and brought mom’s phone with me, and then I called my uncle and asked him to come and sort things out, ‘cause I was really scared…. (p. 6).

Similarly, of the children in Chanmugam’s (2015) study, eight report collaborating with family members such as siblings, grandmothers, and aunts to escape DVA. This involved leaving the home with a family member, calling police or other external agencies, or hiding within the home. Anthony (age 13) reports that he and his brothers: “…went in one room and stayed together…We would go in one of our rooms and lock the door” (p. 110).

In many cases, the actual escape from the violent situation happened suddenly, possibly after planning by the mother. In Bowyer and colleagues’ (2015) analysis of data collected from five girls aged 10–16 in the UK in temporary accommodation with their mothers, Rhiannon says:

I think he [perpetrator] went out somewhere…just quickly like we went to the downstairs ‘cause we lived on like the top floor…so, quickly went to the middle floor to see if he was going to come up in the lift or not and then we called the taxi and we waited until he came and then we ran outside to the taxi and then we went here [refuge]. (p. 308)

Residence in DVA refuges or shelters is explored in a small number of papers, though it is evident from children’s accounts that their removal from a violent home by no means brought an end to their problems. Life in a shelter can be challenging, and the sense of powerlessness over circumstances that is a feature of DVA can continue long after families have been moved to a refuge. Överlien’s (2011a) respondents (aged 11–17) describe negative aspects of shelter life such as its rigid rules, having to keep its location secret and restrictions on visitors.

#### Managing relationships postseparation

This smaller theme—strongly related to the turning points and transitions theme—is about the continuation of DVA, in some form, after parental separation (often in the context of fathers’ contact with children) and the ways in which children struggle to manage relationships in the postseparation period.

Morrison (2015) highlights several instances of ongoing abuse in her analysis of data collected from 18 children (8–14 years old) in Scotland who continued to see nonresident fathers after separation. Michael, for example, says that “…sometimes my dad says not very nice things to my mum when I am going into my dad’s car which makes me feel upset…He said my mum is an idiot” (p. 279). Children also report problems managing relationships with siblings after DVA, as well as their parents. Tanya, a young person in a focus group in Stanley et al. (2012) says that “When me and my brother was younger, my dad used to like hit us and my dad’s left now, my brother’s started” (p. 195).

#### Impact of DVA

Very few studies focus specifically on the impacts of DVA; most papers included here have a more general focus. However, it is evident—from a reading of children’s accounts—that violence does affect children’s health and well-being. In the sample, very few children describe physical health impacts (or, where they do, this cannot always be related to DVA) though some children report being injured during acts of violence. The health-related effects of DVA might be evident where children are asked to reflect on their experiences (in therapeutic settings, for example). In Peled & Edleson (1992), an 8-year-old girl relates how, when she thinks about her experiences, she:

…get[s] [a]…picture in my head, and it just all goes black. And then I get teary eyes, but I don’t cry…And then my stomach starts to hurt…my stomach hurts, probably cause I’m nervous…. (p. 335).

Here, we examine two main areas of impact highlighted by children in the sample: pain, loss, and a desire for normality; and sleep and hypervigilance.

##### Pain, loss, and a desire for normality

The emotional consequences of DVA are reported across most studies, with children describing profound feelings of fear, anxiety, and emotional pain during and after DVA: “It was all pain for us. We had to take everything. Mom took a lot, but I mean we had to watch” (Susan, in Bennett, 1991, p. 434). Child respondents often use words and terms to convey a sense of loss or having missed out—on a childhood, on a “normal” family or the chance of a “normal” life—usually because they had to grow up too quickly:

I’ve never actually had that time to live and be a kid, I haven’t had that time to go running about, messing with my friends or…play football…I just used to run away and find that, that little moment where I could sneak out the house and…just hide under trees. (Ann, young people’s focus group 3, Stanley et al., 2012, p. 196)

Given the emotional fallout from DVA, it is not surprising that some authors highlight the difficulties children have in recounting their experiences. In one analysis with children aged 8–14 years (Georgsson et al., 2011), the researchers focus as much on the ways that children speak about their experiences as on the content of their accounts. Bennett (1991) also notes in her study with five adolescent girls that respondents report not always remembering their experiences: “I know I’ve blocked it out but I just can’t seem to remember it…” (Girl, p. 434).

Problems recounting traumatic experiences might be related to the secrecy surrounding violence. In Peled and Edleson’s (1992) discussion of the evaluation of an intervention, disclosing DVA is about on abuse in the children’s families. This, the authors say, “…alludes to the tangible, solid nature of the emotional isolation that many children of battered women appear to experience…” (p. 331).

##### Sleep and hypervigilance

Disrupted sleep patterns—either during or after DVA—were mentioned in 12 of the papers in our focused sample. Sleeplessness might be the result of noises accompanying violence, or increased alertness to the signs of impending violence so that children in contexts of DVA fail to sleep, or sleep badly in order to maintain a watch:

At night time I can’t really sleep because like I feel like he’s going to like come and to like do something or get into the house. (Jackie, young people’s Focus Group 5, Stanley et al., 2012, p. 195).

[O]ne time I came across my eight year-old sister…going round the house checking the gas hobs were off because she thought he’d leave them on at night to burn down the house…it turns out she’d been doing that for about a month, getting up in the middle of the night to check. (Young person, Buckley, Holt, & Whelan, 2007, p. 300)

Poor sleep can be related to a more generalized vigilance or hypervigilance (Ericksen & Henderson, 1992), an idea discussed in several papers where children report an ability to discern signs of imminent violence. Vigilance can give children a “head start” so that they are better prepared to manage DVA or are primed to adopt strategies to save self or others. Hypervigilance can also result in children who are constantly “on edge”: “Living in constant worry about whether your parents are fighting is surely harmful…It’s like being constantly in a state of ‘stand by’” (Avi, male, 15-year-old, in Goldblatt, 2003, p. 541).

#### Children’s expressions of hope for the future

This less prominent theme is about how children hope for the future. These children often express a belief in being able to transition from a violent present or past to a safer, more hopeful future. As Överlien (2011b) observes, the future that such children imagine is one that would seem quite “ordinary” to most people:

…the children are not talking about…“stories one likes to tell,” such as graduating from school, throwing birthday parties or winning the lottery. They talk about what most of us would define as “ordinary life” i.e. a life with school, friends, jobs for the adults and pets. These are all parts of life the majority of us take for granted. (p. 11)

Children may draw on religious or spiritual beliefs to help them make sense of the past and imagine a more hopeful future. While religious or spiritual beliefs were mentioned by respondents in several papers, only one (Benavides, 2012) specifically focuses on the role of spirituality among children (most of the 14 adolescent participants are female and most have Hispanic or African American family backgrounds) affected by DVA. In the analysis, many adolescents express a belief in God or a higher spiritual power and a desire to learn from the past. Half of the young respondents in Benavides’ sample identify their beliefs as a source of help. For Brian, believing in a higher power helps him feel less alone in the world; his faith is a: “…shield…protecting you, comforting you, keeping you warm, keeping you alive…somebody is there for me” (p. 169).

## Discussion

This article reports findings from a synthesis of qualitative literature on children’s experience of DVA. From the 33 published papers, six overarching themes were identified. Children and young people relate powerful accounts of the everyday, lived reality of DVA in many forms. Children also provide accounts of coping against the odds (in often materially deprived environments), responding strategically and creatively to violence, and demonstrating maturity and protectiveness toward others (e.g., mothers, siblings, animals). DVA means living with pervasive fear and anxiety, with a sense of loss and living a childhood which is far from “normal.” Other themes focused on negotiating turning points and transitions after their mother has exited the abusive relationship. This body of work also points to myriad ways in which children reflect on their experiences to identify hope for the future.

These themes resonate strongly with those in Hines’s (2015) systematic review of qualitative studies focused on coping strategies and service needs. Hines’s themes—*paths to coping and resilience, hope for the future and wisdom*—are analogous to the *children’s expressions of hope for the future* theme we identified. The *cultivating connections* theme is similar to our *turning points and transitions* theme. VOICES’ findings are also similar to those presented in Ravi and Casolaro (2018). *Context of the abuse* in their review is analogous to our *lived experience of DVA* here, and their *immediate reactions to exposure to the abuse* is similar to *children’s agency and coping* in VOICES.

### Review Strengths and Limitations

VOICES utilized systematic review methodology with explicit inclusion and exclusion criteria, a comprehensive search, quality assessment of papers, and a transparent synthesis of the data. We also used scoping searches to inform development of our text word and indexing search terms. Below we highlight possible limitations.

It is possible that relevant studies have been missed at the search stage. We did not conduct a sensitivity analysis of our themes just using the higher quality. At Stage 2, we only included papers that presented enough *verbatim* text for meaningful analysis; we recognize this is, to some degree, a subjective criterion. The exclusion of studies not reported in peer-reviewed journal papers might have limited the final synthesis. Finally, as with all qualitative research, no claims as to the generalizability of findings can be made here. However, VOICES did draw on a large sample (33) of relatively rich accounts from children, and our themes are consistent with those emerging in other reviews of qualitative studies.

A key limitation here, which we share with other reviews, is that all the included papers reported analyses of data collected from children living in shelters or refuges or already accessing services. The voices of children experiencing ongoing DVA at home, and those who have never sought professional support, are always invisible.

### Recommendations

The results of the VOICES study, along with those from related studies like IMPROVE (Howarth et al., 2016; Howarth et al., 2019), can inform both the research agenda in this field to better articulate the needs of children experiencing DVA and guidance to professionals on how best to respond to those needs. Here, we make two broad recommendations for professionals delivering services to these children.

First, all professionals interacting with children impacted by DVA should be mindful of the diversity in children’s experiences of DVA. Listening carefully to children’s own accounts is likely be more effective than an assumption that children from violent homes are affected in the same way. Children can struggle to articulate their experiences, which may be related to the severity of the violence, and they may sense that DVA should remain secret or fear that they will not be believed, so are likely to need time and space to communicate their experiences to professionals. Practitioners should also be aware that readiness to communicate is likely to be affected by a child’s developmental stage.

Second, VOICES found that children and young people describe responding strategically to DVA, often in diverse ways, many of which involve protection of self and others or being innovative. Children might have mixed feelings about their actions and may need the opportunity and space to process these with professionals.

**Table 3.** Implications for Practice, Policy, and Research

We recommend that professionals be mindful of diversity in children’s experiences of DVA and tailor their treatment approach accordingly. Listening carefully to children’s own accounts may be more effective than an assumption that children are affected by DVA in the same way. Children and young people describe responding strategically to DVA; children might have mixed feelings about this and may need the opportunity to explore this with professionals.

While there are several reviews on children’s experiences of DVA, this is the first large-scale, systematic review of the qualitative research that draws solely on children’s perspectives and that did not limit by publication year, age, or other study characteristics (except language). We believe this review adds a significant new dimension to an understanding of the impact of DVA on children. Most of the research previously reviewed, because of its quantitative nature and focus on a limited number of outcomes, provides a useful, but partial, analysis of children’s experiences. Qualitative reports drawing on children’s own accounts present a deeper, more complex, and often more nuanced picture of children’s experiences. In particular, children’s actions during DVA, their resourcefulness in responding to violence, and their capacity for meaning-making and expressions of hope for the future are aspects of their experience highly suited to investigation using a qualitative methodology. Read alongside the many quantitative reviews, VOICES will help practitioners gain a more rounded understanding of children’s experiences, so that treatment approaches are more appropriately tailored to children’s needs.
